# Electrophoretic Analysis of Indian Isolates of *Mycoplasma agalactiae* and *Mycoplasma bovis* by SDS-PAGE and Immunoblotting

**DOI:** 10.1155/2014/892421

**Published:** 2014-04-03

**Authors:** Amit Kumar, N. C. Srivastava, V. P. Singh, Jai Sunder

**Affiliations:** ^1^Department of Microbiology, Uttar Pradesh Pandit Deen Dayal Upadhayay Pashu Chikitsa Vigyan Vishwa Vidyalaya Evam Go-Anusandhan Sansthan (DUVASU), Mathura 281001, India; ^2^National Referral Laboratory on Mycoplasma, Division of Bacteriology & Mycology, Indian Veterinary Research Institute, Izzatnagar 243122, India; ^3^Division of Bacteriology & Mycology, Indian Veterinary Research Institute, Izzatnagar 243122, India; ^4^Divisionof Animal Science, Central Agricultural Research Institute, Port Blair, A & N Islands 744101, India

## Abstract

*Mycoplasma agalactiae* and *Mycoplasma bovis* both are responsible for respiratory conditions in sheep and goats. *M. agalactiae* is a major pathogen of sheep and goats and accounts for almost 90% of outbreaks of contagious agalactia syndrome in goats and almost 100% in sheep. On the basis of clinical signs and cultural, morphological, and biochemical characterization it is almost impossible to differentiate between both the species. Moreover, due to presence of genomic and proteomic similarity most of the time routine diagnostic tests fail to differentiate between them. Hence the present study was conducted to find out the protein profile of isolates of both the species by SDS-PAGE and to find out the cross-reacting as well as differentiating immunogenic proteins by Immunoblotting, which can be of immunoprophylactic as well as diagnostic values. The study revealed 6-7 major immunogenic cross-reactive proteins with the presence of two important non-cross-reacting species specific polypeptides particularly 25.50 and 24.54 kDa in *M. agalactiae* and *M. bovis*, respectively, that might be of diagnostic values.

## 1. Introduction 

Mycoplasmas are the smallest, simplest, and self-replicating organisms with the minimum set of organelles required for the growth and replication. Till now more than one hundred species of mycoplasmas [1, 2], widespread in nature as pathogens of human, animals, birds, fishes, reptiles, arthropods, and plants, have been recognized [1–3]. Out of these species* Mycoplasma agalactiae* and* Mycoplasma bovis* both are important pathogens of small ruminants [3–5].* M. agalactiae *accounts for almost 90% of outbreaks of contagious agalactia syndrome in goats [6] and almost 100% in sheep [4, 7]. Contagious agalactia causes high economic losses due to loss in milk yield and kids/lambs because of abortions, neonatal deaths, and loss of animals [4, 8, 9]. In contrast to* M. agalactiae*,* M. bovis* is supposed to be a major pathogen in calf pneumonia complex and the isolation rates of it have been reported in the range of 23 to 35% [2, 10, 11]; however recently it has been observed in the respiratory infection of sheep with significant mortality [3].* M. agalactiae* and* M. bovis* are two closely related species [11, 12] and were earlier regarded As subspecies of the same species [11]. Moreover, it is difficult to differentiate* M. agalactiae* and* M. bovis* on the basis of morphological, biochemical, and traditionally used serological tests due to many common antigens and receptors on cell surface for antibodies [5, 11–13]. Hence, the present study was designed to identify the non-cross-reactive as well as cross-reacting protein antigens of both the species as a prospective antigen for their detection through serological tests.

## 2. Material and Methods 

### 2.1. Mycoplasma Strains

Two strains of* M. agalactiae* RPNS 216 and RPNS 200 isolated from pneumonic goats and one strain of* M. bovis* NC 317 were used in the present study.

### 2.2. Culture Media

The modified beef horse serum liquid (MBHS-L) medium for the propagation of* M. agalactiae* strains and liquid B medium for the revival and propagation of* M. bovis* strain were prepared according to the standard method [10]. For the colony characteristics solid media were prepared by the addition of 1.2% Bacto Agar (Difco) in respective liquid medium [10].

### 2.3. Whole Cell Antigens (WCA)

WCA were prepared as per earlier prescribed method [14] with slight modifications. Actively growing 2 to 5 mL of* M. agalactiae* and* M. bovis* culture was inoculated in 10 mL liquid medium and incubated at 37°C for 48 hours. The growth was confirmed by the change in pH (change of color red to yellowish orange). This growth was subsequently transferred to larger volume of media and incubated for 4 to 5 days to obtain sufficient growth. Simultaneously the growth was checked for purity on Robertson Cooked Meat (RCM), Sabouraud's Dextrose Agar (SDA), and Blood Agar media. The growth was centrifuged at 10,000 rpm for 25 minutes using a refrigerated centrifuge (Sorvell, RC-5C). The pellets were washed thrice with PBS (pH 7.2) and finally suspended in 10 mL of PBS (pH 7.2). The protein concentrations of WCA were estimated by the standard method [15].

### 2.4. Sonicated Supernatant Antigen (SSA)

Sonicated antigens of all the isolates were prepared from the whole cell antigen by the method described earlier [16] with slight modification. For the preparation of SSA from WCA, the whole cell antigens were diluted in PBS (pH 7.2) and sonicated with MSE-Soniprep 150 at 10 microns by applying 10 jerks, each of 90 seconds with the interval of 30 seconds, on ice. Thus prepared sonicated antigens were further centrifuged at 13,000 rpm for 30 minutes at 4°C (Biofuge, Fresco). The supernatant was separated carefully and was used as SSA. The protein concentration of SSA was estimated by the previously described procedure [15].

### 2.5. Production of Hyperimmune Sera

To assess the immunogenicity of WCA and SSA polypeptides through Immunoblotting, the polyclonal hyperimmune serum was raised against* M. agalactiae* (RPNS 216) and* M. bovis* (NC 317) in white New Zealand rabbits (obtained from LAR, IVRI, Izatnagar), according to the standard protocol [17]. These antisera were tested by slide agglutination test [18] and titer was estimated by indirect haemagglutination (IHA) [19] with slight modification [20]. Sera were finally filtered through 0.20 *μ*m filter (Sartorius) and stored at −20°C for further use.

### 2.6. Sodium Dodecyl Sulphate Polyacrylamide Gel Electrophoresis (SDS-PAGE)

The SDS-PAGE of both (WCA and SSA) of* M. agalactiae* (RPNS 216 and RPNS 200) and* M. bovis* (NC 317), under denaturating, reducing conditions, was performed by the method of Lammli [21] with recommended modifications [14]. Electrophoresis was carried out using 12.5% separating and 4% stacking gel at 50 volts for 1 h and at 100–110 volts afterwards till the end of run. The determination of molecular weights was based on the distance migrated by the polypeptides in the gels in comparison to the distance migrated by polypeptide markers of known molecular weights (Biored) [22].

### 2.7. Immunoblotting

The WCA and SSA of all the isolates separated on 12.5% (w/v) SDS-PAGE slabs [21] were transferred electrophoretically on nitrocellulose membrane papers (NCP) (Sartorius). Then these membranes were cross-blotted with the hyperimmune serum raised against* M. agalactiae* (RPNS 216) and* M. bovis* (NC 317) separately by the previously described method [23] to find out cross-reactive polypeptides.

## 3. Results

**Figure 1:**
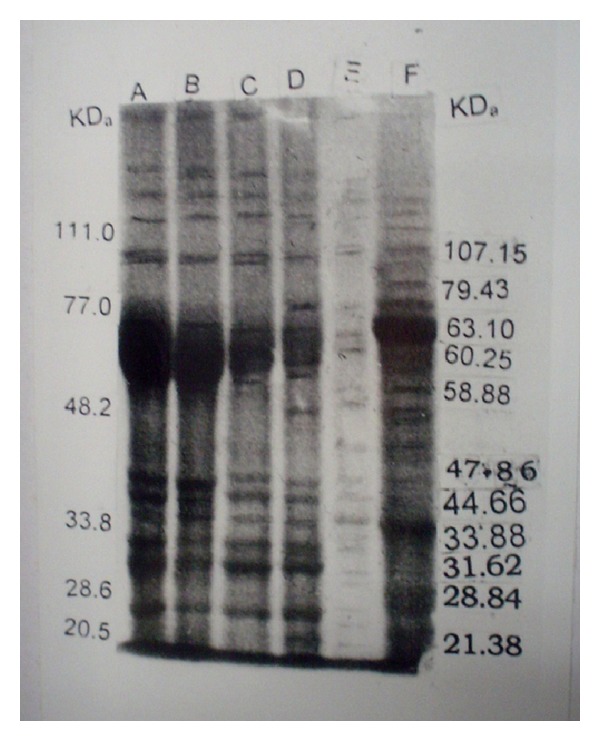
SDS-PAGE analysis of whole cell and sonicated supernatant antigens. Lane A: WCA of* M. agalactiae* RPNS 216. Lane B: WCA of* M. agalactiae* RPNS 210. Lane C: SSA of* M. agalactiae* RPNS 216. Lane D: SSA of* M. agalactiae* RPNS 210. Lane E: SSA of* M. bovis* NC 317. Lane F: WCA of* M. bovis* NC 317.

**Figure 2:**
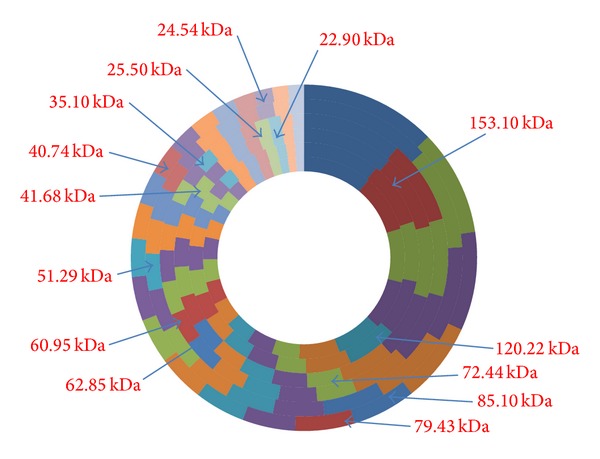
SDS-PAGE protein profiles have been depicted centripetally from* M. agalactiae* (RPNS 216) WCA;* M. agalactiae* (RPNS 216) SSA;* M. agalactiae* (RPNS 210) WCA;* M. agalactiae* (RPNS 210) SSA;* M. bovis* (NC 317) WCA;* M. bovis* (NC 317) SSA.

The results of SDS-PAGE profiles of the two isolates of* M. agalactiae* and one of* M. bovis* have been presented in Figure 1. The molecular weights of the polypeptides in all the isolates ranged between 181.97 and 20.89 kDa (Figure 1). The number of polypeptides varied isolate to isolate and type of antigens used for SDS-PAGE (Figure 2). Both the isolates of* M. agalactiae* revealed 24 and 25 polypeptides in WCA and SSA, respectively, whereas WCA and SSA of* M. bovis* isolate revealed only 21 and 22 polypeptides, respectively. The patterns of major polypeptides were almost similar with minor differences. Profile of* M. agalactiae* isolates showed the difference of two polypeptides; the polypeptide of 120.22 kDa was present in only SDS-PAGE profile of* M. agalactiae* RPNS 216 whereas polypeptide of 62.85 kDa was present in the profile of* M. agalactiae* RPNS 210 (Figures 1 and 2). The polypeptide of 35.10 kDa was present only in the SSA of both the isolates of* M. agalactiae* (Figures 1 and 2).

The SDS-PAGE profiles of* M. bovis* isolate also produced similar protein profile with almost similar migration patterns. However, the number of polypeptides appeared lesser in number (Figure 1) with 21 and 22 polypeptides in WCA and SSA, respectively (Figure 2). The polypeptide of 79.43 kDa was present only in SSA of* M. bovis*.

The polypeptides of molecular weights 153.10, 72.44, 60.95, 41.68, 25.50, and 22.90 kDa were present only in WCA and SSA of both the isolates of* M. agalactiae.* The polypeptides of 122.22, 62.85, and 35.10 kDa were absent from the protein profile of the* M. bovis* and were present exclusively in respective antigens of* M. agalactiae* (Figures 1 and 2). Moreover, the isolate of* M. bovis* was having exclusive polypeptide of 85.10, 51.29, 40.74, and 24.54 kDa along with 79.43 kDa in SSA only (Figures 1 and 2).

**Figure 3:**
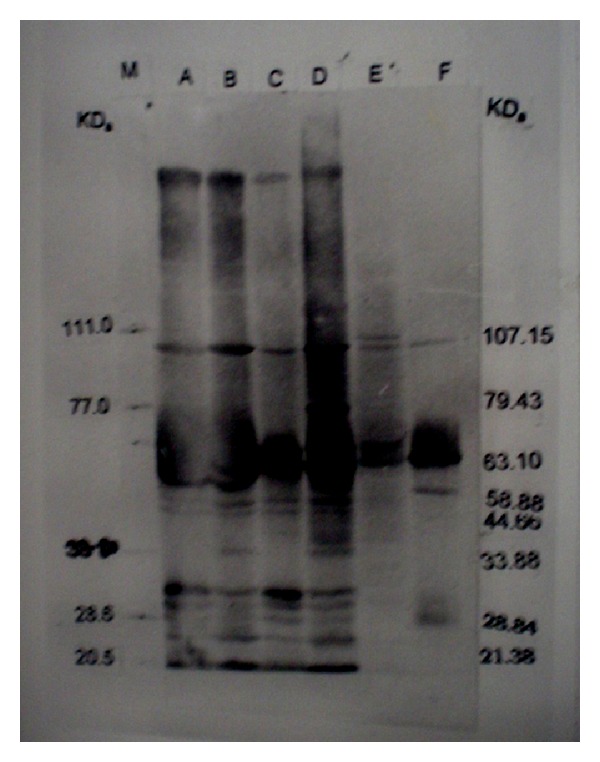
Immunoblot analysis of whole cell and sonicated supernatant antigens against* M. agalactiae* RPNS 216 hyperimmune sera. Lane A: WCA of* M. agalactiae* RPNS 216. Lane B: WCA of* M. agalactiae* RPNS 210. Lane C: SSA of* M. agalactiae* RPNS 216. Lane D: SSA of* M. agalactiae* RPNS 210. Lane E: SSA of* M. bovis* NC 317. Lane F: WCA of* M. bovis* NC 317. Lane M: Molecular weight marker.

**Figure 4:**
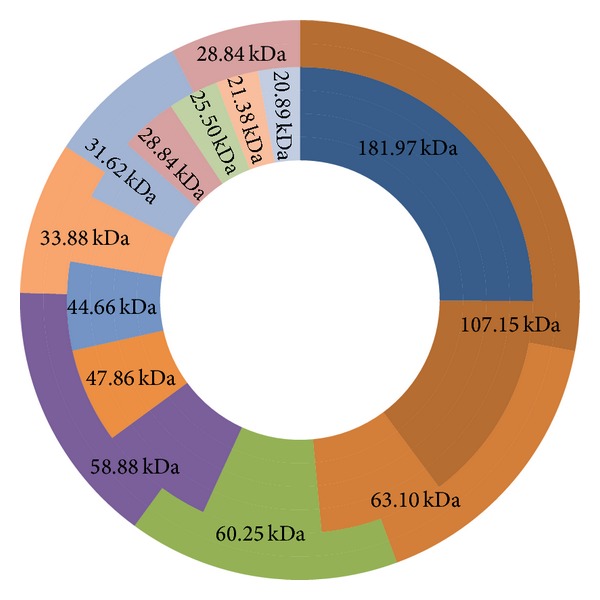
Immunoblot analysis depicted centripetally from* M. agalactiae* (RPNS 216) WCA;* M. agalactiae* (RPNS 216) SSA;* M. agalactiae* (RPNS 210) WCA;* M. agalactiae* (RPNS 210) SSA;* M. bovis* (NC 317) WCA;* M. bovis* (NC 317) SSA.

**Figure 5:**
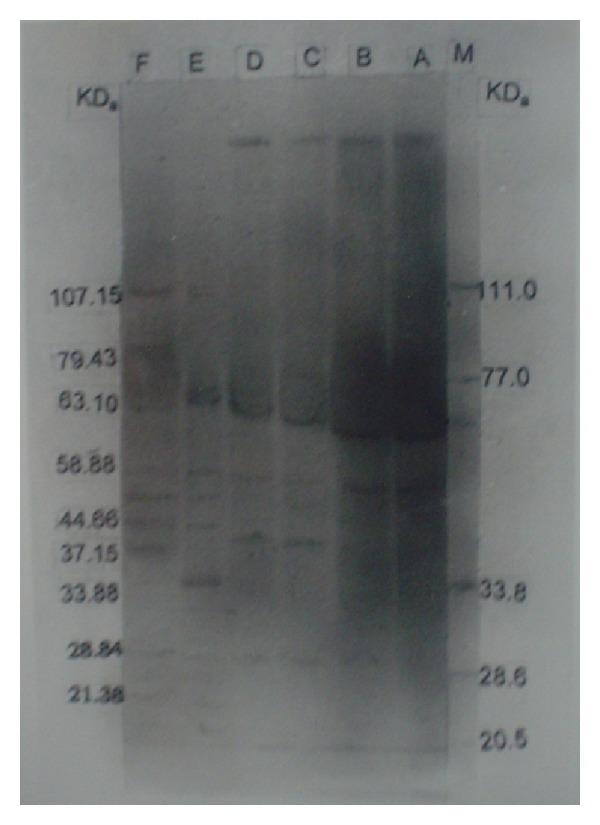
Immunoblot analysis of whole cell and sonicated supernatant antigens against* M. bovis* NC 317 hyperimmune sera. Lane A: WCA of* M. agalactiae* RPNS 216. Lane B: WCA of* M. agalactiae* RPNS 210. Lane C: SSA of* M. agalactiae* RPNS 216. Lane D: SSA of* M. agalactiae* RPNS 210. Lane E: SSA of* M. bovis* NC 317. Lane F: WCA of* M. bovis* NC 317. Lane M: molecular weight marker.

**Figure 6:**
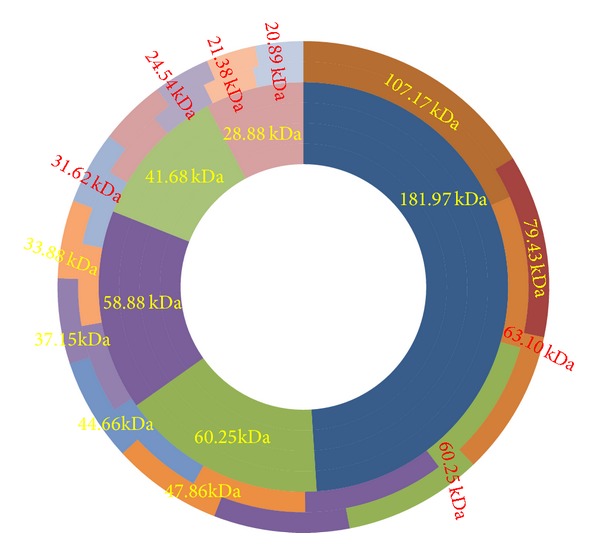
Immunoblot analysis depicted centripetally from* M. agalactiae* (RPNS 216) WCA;* M. agalactiae* (RPNS 216) SSA;* M. agalactiae* (RPNS 210) WCA;* M. agalactiae* (RPNS 210) SSA;* M. bovis* (NC 317) WCA;* M. bovis* (NC 317) SSA.

When Immunoblotting was performed with the hyperimmune serum against* M. agalactiae* RPNS 216 of* M. bovis* NC 317 the immunoblots revealed almost similar patterns of immunogenic polypeptides with the difference of some immunogenic polypeptides. As usual isolates revealed more immunogenic polypeptides with homologous hyperimmune serum, namely, 13 each in* M. agalactiae* isolates (Figures 3 and 5) and 13 and 14 in WCA and SSA of* M. bovis* (Figures 5 and 6). The numbers of cross-reactive antigens were comparatively lesser with 5 numbers of polypeptides in* M. agalactiae* isolates (Figures 5 and 6) and 7 in* M. bovis* isolate (Figures 3 and 4). The immunoblot profiles of both the isolates of* M. agalactiae* were identical with both homologous and heterologous hyperimmune serum (Figures 4 and 6). The immunogenic polypeptides of molecular weights 47.86, 44.66, 33.88, 31.62, 21.38, and 20.89 were present in immunoblot of all the isolates but only with homologous hyperimmune serum (Figures 3, 4, 5, and 6). Only two polypeptides one each in* M. agalactiae* and* M. bovis* 25.50 and 37.15 kDa, respectively, were present exclusively in immunoblots of* M. agalactiae* and* M. bovis* with homologous hyperimmune serum (Figures 3, 4, 5, and 6).

## 4. Discussion

The immunologic cross-reactivity among members of the mycoplasmatales is well documented and tests, namely, immunodiffusion, agglutination, two-dimensional immunoelectrophoresis, counter current immunoelectrophoresis, ELISA, immunobinding assay, polyacrylamide gel electrophoresis [1, 5, 11, 12, 24], and variety of other methods with the use of polyclonal serum, failed due to many common antigens and receptors on cell surface for antibodies [1, 5, 13]. The results of the present study are also in the concurrence of previous findings revealing many cross-reactive polypeptides [5, 11, 12, 24]. Moreover 52 and 60 common polypeptides were observed in almost similar range and pattern in* M. agalactiae* [25] and* M. bovis* [26, 27] with the presence of 5 to 7 major proteins in the range of 90 to 20 kDa. Similarly almost identical patterns with minor differences were recorded in* M. agalactiae* and* M. bovis* with one-third polypeptides of identical molecular masses [12]. However, in an earlier report, no common protein band but areas of homology did exist among different mycoplasmal species including* M. agalactiae* and* M. bovis* [13].

Based on previous information identification of species specific immunogenic proteins is always of diagnostic as well as prophylactic values. For that Immunoblotting, a sensitive and specific confirmatory test for the identification and isolation of immunogenic proteins was performed with homo- and heterologous hyperimmune serum [23]. The presence of 5–7 major immunogenic proteins (Figures 3, 4, 5, and 6) obtained during the Immunoblotting might be of diagnostic as well as protective values. These findings are in the concurrence of many earlier reports in various species of mycoplasmas* M. ovipneumoniae* [26],* M. mycoides* cluster [28],* M. hominis* [29],* M. hyopneumoniae* and* M. flocculare* [22],* M. agalactiae* [12, 14, 30], and* M. bovis* [12, 31, 32]. As protein patterns obtained by SDS-PAGE provide an indirect measure of the coding capacity of mycoplasmal genome and comparison sought to provide an approximate reflection of probable genomic capacity. However, each band may consist of many proteins of similar molecular weight as separation using SDS is dependent on molecular weight only [33]. Hence the presence of six to seven major immunogenic polypeptides in all the three isolates of both the species particularly the polypeptides of 60.25 and 28.84 kDa can be of immunoprophylactic use against both the species. Similarly the presence of species specific polypeptides particularly 25.50 and 24.54 kDa in* M. agalactiae* and* M. bovis,* respectively, might be of diagnostic values. However to authenticate the findings studies with more number of isolates from different geographical region are required.

## 5. Conclusions

It can be concluded from the present study that both the isolates of* M. agalactiae* have almost similar protein profile and presence of identical immunogenic polypeptides. Moreover, protein profile of* M. agalactiae* and* M. bovis* are almost similar with the presence of many cross-reactive major polypeptides. These can be differentiated by the presence of certain species specific immunogenic proteins or by using the monoclonal antibodies raised against those proteins in the diagnostics. However, a large number of isolates are required to be examined before these conclusions are put to practice.
